# Storytelling in schizophrenia: Neuropsychological basis of pragmatic language dysfunction (preliminary study)

**DOI:** 10.1192/j.eurpsy.2021.1394

**Published:** 2021-08-13

**Authors:** I. Gornushenkov, I. Pluzhnikov

**Affiliations:** 1 Department Of Endogenous Mental Disorders And Affective States, The Mental Health Research Center, Moscow, Russian Federation; 2 Department Of Adult Neuropsychology And Abnormal Psychology, Moscow Institute of Psychoanalysis, Moscow, Russian Federation; 3 Department Of Youth Psychiatry, The Mental Health Research Center, Moscow, Russian Federation

**Keywords:** Pragmatic function of speech, schizophrénia, neuropsychology, Storytelling

## Abstract

**Introduction:**

Storytelling could be considered as one of the ecological way to study the pragmatic function of speech in schizophrenia. It demands the ability to create narrative (text) that would be appropriate to particular context. Neuropsychological basis of text-context relation impairment in schizophrenia needs clarification.

**Objectives:**

To study neuropsychological correlates of pragmatic text-context impairment revealed during storytelling in patients with schizophrenia.

**Methods:**

Participants were 14 inpatients with schizophrenia and 18 students without mental disorders. Neuropsychological functioning was measured in both groups according to Luria’s method. Pragmatics assessed by storytelling on images which simultaneously depicts some narrative that should be correctly decoded and after expressed to investigator. The images were taken from Luria’s neuropsychological album and Bidstrup’s drawings.

**Results:**

Stories of patients with schizophrenia were different from control stories in two ways. Some patients produced stories which predominantly characterized by incompleteness that don’t give an opportunity to understand their narratives as connected whole because of its lacunarity. In other cases, stories predominantly characterized by distortion of the storyline which became not realistic and don’t match with the original picture. Incompleteness errors primarily correlates with neuropsychological dysfunction of left frontal lobe (p<0,001). Distortion errors also mainly correlates with dysfunction of frontal lobes (p<0,01), but qualitative analysis reveals right hemisphere involvement.

**Conclusions:**

Impairment of the pragmatic function of speech during storytelling in schizophrenia could manifest itself in at least two different ways. Preliminary results show that it could be connected with different neuropsychological mechanisms and worth considered with left-right frontal lobes opposition.

**Conflict of interest:**

The reported study was funded by RFBR, project number 20-013-00772

